# A New Feature Descriptor for Multimodal Image Registration Using Phase Congruency

**DOI:** 10.3390/s20185105

**Published:** 2020-09-08

**Authors:** Guorong Yu, Shuangming Zhao

**Affiliations:** School of Remote Sensing and information Engineering, Wuhan University, No. 129, Luoyu Road, Wuhan 430070, China; ygrong@whu.edu.cn

**Keywords:** feature matching, phase congruency, log-Gabor filter

## Abstract

Images captured by different sensors with different spectral bands cause non-linear intensity changes between image pairs. Classic feature descriptors cannot handle this problem and are prone to yielding unsatisfactory results. Inspired by the illumination and contrast invariant properties of phase congruency, here, we propose a new descriptor to tackle this problem. The proposed descriptor generation mainly involves three steps. (1) Images are convolved with a bank of log-Gabor filters with different scales and orientations. (2) A window of fixed size is selected and divided into several blocks for each keypoint, and an oriented magnitude histogram and the orientation of the minimum moment of a phase congruency-based histogram are calculated in each block. (3) These two histograms are normalized respectively and concatenated to form the proposed descriptor. Performance evaluation experiments on three datasets were carried out to validate the superiority of the proposed method. Experimental results indicated that the proposed descriptor outperformed most of the classic and state-of-art descriptors in terms of precision and recall within an acceptable computational time.

## 1. Introduction

Image registration is a vital step in many computer vision tasks, such as three-dimensional (3D) image reconstruction, image stitching, super resolution, and medical image processing. Methods for image registration can be categorized into two types: feature-based methods and area-based methods [1,2]. Feature-based methods focus on the local information around the features (for example: lines, corners, and areas) and use this information to find the correspondence in the feature space. Area-based methods, also called template matching, utilize a local window to find the correspondence according to some similarity metrics such as sum of squared difference (SSD), normalized cross-correlation (NCC), and mutual information (MI). However, both of these types of traditional methods cannot be applied to multimodal image registration because of the non-linear tone mappings in multimodal images. For example, scale invariant feature transform (SIFT) [3,4] is widely used in image registration, as it can correctly describe the local information around the feature points. However, the non-linear tone mappings in multimodal images lead to different feature descriptors of the correct corresponding keypoints according to the histograms of the gradients in the local windows, thereby yielding unsatisfactory results. To solve this problem, we developed a new feature descriptor that is invariant to non-linear tone mappings.

As to feature-based methods, SIFT is the most popular one among them. Since it was first proposed, SIFT has been widely used in many computer vision tasks because of its invariance to scale change and its distinctiveness. Furthermore, many SIFT-like methods, such as principal component analysis SIFT (PCA-SIFT) [5], speeded up robust features (SURF) [6], gradient location and orientation histogram (GLOH) [7], and affine-SIFT (ASIFT) [8], have been developed to improve its performance. The histogram of oriented gradients (HOG) [9] is also a widely used feature descriptor in computer vision and image processing; the essential thought behind the HOG descriptor is that the local object appearance and shape within an image can be described by the distribution of the intensity gradients or the edge directions. However, SIFT-like methods and HOG are designed for mono-spectral image pairs (e.g., RGB–RGB); that is, they can only be applied to images with the same modality. Images with different modalities usually have non-linear tone mappings, and this will yield an inconsistent orientation assignment and different descriptors of correct correspondences if the descriptor generation is based on the histogram of gradients. Thus, these methods will fail in these cases. In the past, researchers have proposed some binary descriptors, such as binary robust independent elementary features (BRIEF) [10], oriented FAST and rotated BRIEF (ORB) [11], and fast retina keypoint [12]. Although these new local features improve the computational efficiency, they are vulnerable to complex radiometric changes. To tackle this problem, many new methods have been proposed; they improve descriptors from the following four aspects: the oriented gradient descriptor generation process is improved; some descriptors are designed on the basis of the distribution of the edge points or the geometric layout; information in the frequency domain is utilized to obtain the descriptors; and deep learning approaches are exploited to tackle this problem.

Researchers have made improvements on the oriented gradient descriptor generation. Chen et al. [13] proposed a descriptor named partial intensity invariant feature descriptor (PIIFD), which can be partially invariant to intensity changes; it can be successfully utilized in multimodal retinal images. Firmenich et al. [14] modified the original SIFT descriptor by reversing the gradient direction, which was out of the range of [0, 180]; they named the resulting descriptors gradient direction invariant SIFT (GDISIFT). This could reduce the influence of the direction reversal in the gradient direction to some degree. Similarly, Saleem [15] improved SIFT by using normalized gradients as the descriptors of keypoints, which increased the distinctiveness to some degree.

Some edge-based and shape-based features have also been proposed. Aguilera et al. [16] extracted the edges of features and transformed curves into a histogram of edges to describe the features, which is named edge-oriented histogram (EOH). Similarly, a local energy-based shape histogram (LESH) [17] is a proposed image descriptor based on the underlying shape, which can be used in medical image processing. A local self-similarity descriptor was first proposed by Shechtman [18] and then improved by Chatfield [19]. This descriptor focuses not on the information formed by pixels but on the geometric layout formed by the patches in a region. It describes the geometric layout information in a region, and thus, it can somehow avoid the problems caused by the non-linear mappings between multimodal images. Ye et al. [20] utilized a block-wise strategy to extract evenly distributed features and then exploited a modified local self-similarity descriptor to represent each feature. This method achieves satisfactory results and improves the accuracy of multimodal image registration to some degree. Similarly, Ye [21] developed a novel descriptor named dense local self-similarity to represent the features in optical and synthetic aperture radar images. It yields more satisfactory results than the NCC and MI methods by extracting the shape property around each feature. Chen et al. [22] proposed a feature descriptor named robust center-symmetric local ternary pattern-based self-similarity descriptor. Its main idea is a rotation invariance description strategy on the local correlation surface. Sedaghat [23] proposed a distinctive order based self-similarity descriptor (DOBSS) for multi-sensor remote sensing image matching. They made some modifications to the orientation assignment and exploited the self-similarity to describe the geometric layout of the features. Moreover, many other different modifications [24,25,26,27,28,29] were made to the self-similarity descriptors to improve the robustness against the non-linear tone mappings between multimodal images.

In addition to the descriptor in the spatial domain, more information can be obtained by convolving the images by using multi-scale and multi-oriented Gabor-based filters, including the Gabor filters and log-Gabor filters. The log-Gabor filter responses are invariant to illumination changes [30], and the multi-oriented magnitudes transmit useful shape information. Aguilera et al. [31] convolved images with log-Gabor filters and then utilized the magnitude of the filter response to describe a feature, which is named log-Gabor histogram descriptor (LGHD). This method assumes that high-frequency response will be robust to different non-linear intensity variations. Kovesi [32] proposed a measure of phase congruency that is independent of the magnitude of the signal, making it invariant to variations in image illumination or contrast. Therefore, a number of methods have been proposed on the basis of the phase congruency. The phase congruency and edge-oriented histogram descriptor (PCEHD) [33] was proposed by combining spatial information (EOH) and frequency information (the amplitude of the log-Gabor coefficients). The histogram of oriented phase congruency (HOPC) was developed and applied for template matching in multimodal remote sensing images [34,35].

In the past few years, deep learning methods have been exploited to align multimodal image pairs [36]. Aguilera [37] presented a novel Convolutional Neural Network (CNN) based architecture, referred to as Q-Net, to learn local feature descriptors that can be applied for matching image patches from two different spectral bands. Cheng [38] recently used a stacked denoising autoencoder to learn a similarity metric that assesses the quality of the rigid alignment of computed tomography (CT) and magnetic resonance (MR) images. Simonovsky [39] used a CNN to learn the dissimilarity between aligned 3D T1 and T2 weighted brain MR volumes.

As to area-based methods, a similarity metric plays a decisive role in template matching. Common similarity metrics include SSD, NCC, and MI. SSD is probably the simplest similarity metric because it only exploits the intensity information. Therefore, it is sensitive to intensity change and cannot be applied to multimodal image registration. NCC is widely used in normal remote sensing image registration because of its invariance to linear tone mappings. However, there exist non-linear mappings between multimodal images; thus, the NCC method fails in this case. Finally, when non-linear tone mappings are considered, MI is commonly used. MI was originally proposed for image registration [40] and detects correspondences by calculating the statistical dependence between two variables. The statistical dependence must be high when one image is a functional tone mapping of another image (irrespective of whether the tone mapping was linear or non-linear). Although MI accounts for non-linear tone mappings, it is hindered by its computational complexity and is sensitive to the window size in template matching. Matching by tone mappings (MTM) [41] was proposed by Hel-Or with the inspiration of NCC. It can be viewed as a generalization of the NCC for non-linear tone mappings. MTM runs considerably faster than MI and is insensitive to the window size. Nevertheless, when functional mappings do not necessarily exist, MI outperforms MTM [42]. In general, these area-based methods can be applied to normal remote sensing images and yield satisfactory results. Only MI and MTM can be used as similarity metrics when non-linear tone mappings are considered. However, they need further improvement, as they are sensitive to the window size or not robust.

Phase congruency is a dimensionless quantity that is invariant to changes in image brightness or contrast; thus, it provides an absolute measure of significance of feature points [32]. Inspired by this, in this paper, we propose a novel descriptor based on the histogram of the oriented magnitude and the histogram of the orientation of the minimum moment of phase congruency. After images are convolved with a bank of log-Gabor filters, the two parts of the descriptor can be calculated quickly over the local windows as the intermediate results when obtaining the phase congruency. The proposed descriptor not only reflects the distribution of magnitudes along each orientation but also provides an indication of the orientation of the feature, which can provide some common intensity invariant information shared by multimodal image pairs.

The remainder of this paper is organized as follows. Section 2 gives a brief introduction of log-Gabor filters and phase congruency and then proposes the novel descriptor. Section 3 introduces the experimental setup and the evaluation criteria and then presents the experimental results obtained on three datasets. Section 4 discusses and analyzes the results of the experiments. Section 5 presents the conclusions and the recommendations for future work.

## 2. Methodology

### 2.1. Feature Extraction

In our paper, feature extraction was the first step. SIFT is the most widely used method in feature extraction step because of its invariance to scale change. We followed the strategies in SIFT to extract features. Local extrema were identified as the candidate keypoints in the Difference of Gaussians (DoG) pyramid. The DoG pyramid of an image was obtained using the difference in two adjacent scale spaces separated by a constant factor. Once a candidate point was located, it was refined to subpixels and eliminated if it was found to be unstable according to the low contrast and the influence of edge.

### 2.2. Feature Description

After the keypoints were extracted, the next step was to describe them by their local information. As mentioned above, the local information could be referred to as orientations, gradients, or correlation surface in a local window. However, conventional orientations, gradients, and local self-similarities are not sufficiently discriminative in multimodal image registration applications because of their non-linear intensity changes. In the Fourier frequency domain, illumination change or intensity change corresponds to the local energy [32]; in the case of multimodal images, we attempted to find a quantity to describe a local region, which was invariant to intensity changes. Phase congruency was a quantity that could solve this problem. In this section, we mainly introduce two topics: (1) how to calculate the phase congruency and (2) how to utilize the intermediate results of the phase congruency to encode our descriptor.

#### 2.2.1. Phase Congruency

In the spatial domain, the local information distributed in the selected window for descriptor generation is prone to being influenced by non-linear intensity changes. Thus, generating a robust descriptor in frequency domain is a good alternative. As we know, a signal can be transformed into frequency domain with the form $F(u)=|F(u)|e^{-i\phi (u)}$, in which *μ* is the frequency, |F(u)| is the amplitude of this signal, and ϕ(u) represents the phase of this signal. Phase is important in image processing [32], but the phase information ϕ(u) supplied by a Fourier transform can only offer the total phase information; i.e., we cannot use the total phase information to generate our local descriptors. In order to develop a local descriptor that is invariant to intensity changes, we need to obtain the local phase information. Phase congruency offers a good choice: In the intermediate process of the calculation of the phase congruency, we also obtained the local phase and the local energy, which provided us with an inspiration for the development of descriptors invariant to non-linear intensity changes.

Phase congruency is the ratio of the total energy to the overall amplitudes of the local Fourier components, which can be obtained by using the log-Gabor filter responses [32]. In two-dimensional (2D) cases, the 2D log-Gabor filter can be obtained by using a Gaussian function of frequency on a logarithmic scale times a Gaussian function of angular direction, which is defined as follows:

(1) $$LG_{s,o}(\omega ,\theta )=\exp\{-\frac{log{\left(\omega /\omega_{s}\right)}^{2}}{2{\left(log\beta \right)}^{2}}\}\exp-\frac{\left(\theta -\theta_{s,o}\right)}{2\sigma_{\theta }^{2}}$$

where (ω,θ) represents the polar coordinates of an image; s and o are the scale and the orientation of this log-Gabor filter bank, respectively; and ($\omega_{s}$,$\theta_{s,o}$) are the center frequency and the center orientation, respectively. The corresponding filter of the log-Gabor filter in the spatial domain can be achieved by applying the inverse Fourier transform. The real part and the imaginary part of the inverse transform are referred to as log-Gabor even symmetric filter $M_{s,o}^{e}$ and log-Gabor odd symmetric filter $M_{s,o}^{o}$. Given an input image I(x,y), the log-Gabor convolution result can be regarded as a response vector as follows:

(2) $$\left[e_{s,o}(x,y),o_{s,o}(x,y)]=[I(x,y)*M_{s,o}^{e},I(x,y)*M_{s,o}^{o}\right]$$

where $e_{s,o}(x,y)$ and $o_{s,o}(x,y)$ are the responses of the convolution results at scale s and orientation o. The amplitude $A_{s,o}$ and phase $\phi_{s,o}$ at scale s and orientation o are given as follows:

(3) $$A_{s,o}=\sqrt{e_{s,o}{\left(x,y\right)}^{2}+o_{s,o}{\left(x,y\right)}^{2}}$$

(4) $$\phi_{s,o}=atan2(e_{s,o}(x,y),o_{s,o}(x,y)).$$

Considering the influence of noise, Kovesi proposed an improved version of phase congruency:

(5) $$PC(x,y)=\frac{\sum_{o}\sum_{s}W_{o}(x,y)\left\lfloor A_{s,o}(x,y)\Delta \Phi_{s,o}(x,y)-T_{o}\right\rfloor }{\sum_{o}\sum_{s}A_{s,o}(x,y)+\epsilon }$$

where ϵ is a small constant to avoid division by zero and $T_{o}$ is the noise threshold at orientation o. $W_{o}(x,y)$ is the weighting factor for the given frequency spread. $\Delta \Phi_{s,o}(x,y)$ is the phase deviation. ⌊·⌋ denotes that the enclosed quantity is equal to itself when its value is positive or zero otherwise. For more detailed information about these parameters, readers can refer to [32].

#### 2.2.2. Descriptor Generation

In the calculation of the phase congruency, there are many useful intermediate results, such as moments of phase congruency, local energy along orientation o, and overall amplitude along one orientation o. Therefore, in order to make full use of the information in the phase congruency, we propose our joint local descriptor based on the orientation of the principal axis about which the moment of phase congruency is minimized and the histogram of oriented magnitudes; here, the orientation of magnitude is defined by us. We use the overall magnitudes along some direction o to determine the orientation of each pixel in the local windows for the descriptor generation. The orientation of magnitude is defined as follows:

(6) $$bin=\underset{o}{\max}\sum_{s}A_{s,o}.$$

The first part of the descriptors based on the orientation of magnitude can be obtained as follows:

1. A window with fixed size is selected around each extracted keypoint, and the voting bin of each pixel in the selected window is calculated according to Equation (6).
2. The local window around each keypoint is divided into 4 × 4 blocks, and every pixel in the block makes a contribution to a certain bin in the histogram in the corresponding block. As mentioned above, here, ‘certain bin’ is determined by Equation (6) for each pixel. Furthermore, the contribution to each bin by every pixel is set to 1.
3. After obtaining all the histograms in each block, we concatenate the histograms and normalize the feature vector by its L2 norm.

**Figure 1:**
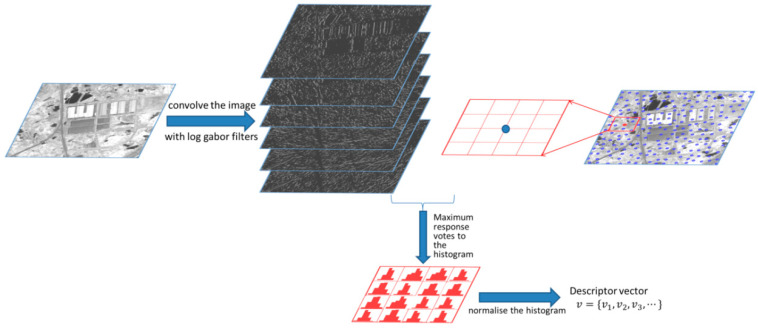
Main steps of how to encode the histogram-oriented magnitude.

Figure 1 presents a brief illustration of how the oriented magnitude-based descriptor is generated.

The second part of the proposed descriptor is based on the orientation of minimum moment of phase congruency. As we can see in Equation (5), the phase congruency is actually calculated along each orientation defined by the log-Gabor filters. For the physically identical keypoints, the variation of the moments with the orientation should be the same. In [43], the principal axis, corresponding to the axis about which the moment is minimized, can provide an indication of the orientation of the feature. Therefore, following the classical moment analysis equations, we compute the following metrics at each point in the image:

(7) $$\begin{array}{c} a=\sum (PC(\theta )cos(\theta ){)}^{2}\\ b=2\sum (PC(\theta )cos(\theta ))(PC(\theta )sin(\theta ))\\ c=\sum (PC(\theta )sin(\theta ){)}^{2}. \end{array}$$

The angle of principal axis O is given as follows:

(8) $$O=\frac{1}{2}atan2(\frac{b}{\sqrt{b^{2}+{\left(a-c\right)}^{2}}},\frac{a-c}{\sqrt{b^{2}+{\left(a-c\right)}^{2}}}).$$

Here, we take O as the orientation of the bin in the second histogram vector. Therefore, the second part of the descriptor can be obtained as follows:

(1) Similar to the first part of descriptor generation, a window of identical size is extracted along each keypoint.
(2) We calculate the angle O for each pixel in the local window and constrain this angle within [0, *π*]. Then, the local window is divided into 4 × 4 blocks, and each block contains a six-bin histogram. Next, we divide O into six identical angle domains: [0, 30], [30, 60], [60, 90], [90, 120], [120, 150] and [150, 180], which correspond to the six bins in each histogram in each block. The contribution of each bin is weighted by the overall amplitudes of each pixel.
(3) After obtaining the four histogram vectors, we concatenate the vectors and normalize them by using the L2 norm. At this step, the generation of the second part of the proposed descriptor is complete.

After the first part and the second part of the descriptor are computed, we concatenate the two descriptors to form the final descriptor with a dimension of 192.

## 3. Experimental Results and Discussion

In this section, the performance of the proposed descriptor is compared with that of SIFT, EOH, PIIFD, LGHD, GDISIFT, PCEHD, HOG, and SURF on three different datasets. The implementations of SIFT, PIIFD, LGHD, EOH, GDISIFT, PCEHD, HOG, and SURF were based on MATLAB code. They were based on publicly available code. The proposed descriptor was also implemented using MATLAB. A detailed description of the datasets is presented in Section 3.1. The evaluation criteria are described in Section 3.2. Parameter settings in our experiments are listed in Section 3.3. A brief description of the compared methods is provided in Section 3.4. The experimental performance evaluation and analysis are discussed in Section 3.5. The computational time analysis is discussed in Section 3.6.

### 3.1. Description of Datasets

**Figure 2:**
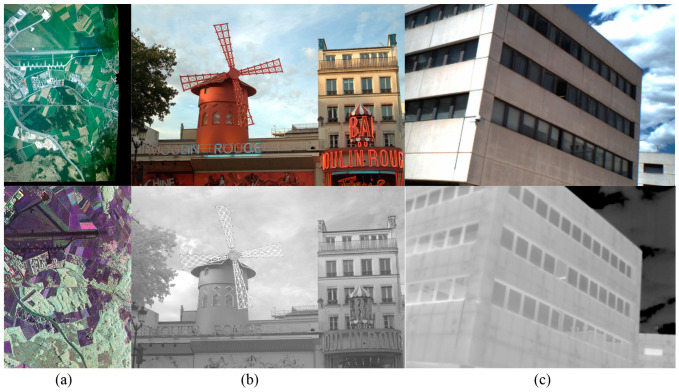
Samples from each dataset. (**a**) The first column pair is from Diaz’s dataset. The second column is from the EPFL dataset, (**b**) in which the second row represents the image captured in the infrared band. (**c**) The third column is from [31], in which the first row represents an RGB image and the second row represents the long wave infrared (LWIR) image from the same scene.

In this study, three types of datasets were collected to test the proposed descriptor. The first type of dataset was chosen from Diaz’s dataset [42]. It contained 15 image pairs of different image sizes, and the modalities taken into account in this dataset were LiDAR intensity, visible, thermal, and depth images. The second type of dataset was from [14], which consisted of 477 images having different sizes, classified into nine categories, and captured in the RGB and near-infrared bands. The scene categories were as follows: country, field, forest, indoor, mountain, old building, street, urban, and water. For this dataset, they were rectified and aligned so that their correspondences should also be found in horizontal lines. The third type of dataset included 44 pairs of RGB–long wave infrared (LWIR) images [31] of different urban scenarios and 100 pairs of visible–LWIR outdoor images [16]. The resolution of the 44 pairs of RGB–LWIR images was 639 × 431, and the resolution of the 100 pairs of visible–LWIR images was 506 × 408. These image pairs were captured using color cameras and infrared cameras. As described in their introductions, all the images were coarsely rectified so that the matches should be found in horizontal lines. Some samples of these three datasets are illustrated in Figure 2.

### 3.2. Evaluation Criteria

In order to quantify the performance of the proposed descriptor, we followed the evaluation criteria used in PCA-SIFT; the four common quantitative evaluation criteria—namely, recall, precision, F-Measure [44], and computational time—were used. In our paper, if the Euclidean distance between a pair of descriptors is smaller than the threshold t, then this pair of descriptors will be termed a match. A correct positive means a match where two keypoints correspond to the same physical location in two images. Similarly, a false positive is a match where the two keypoints come from different physical locations. The recall, precision and F-Measure are defined as follows:

(9) $$Recall=\frac{correct\;positives}{total\;number\;of\;real\;positives}$$

(10) $$Precision=\frac{correct\;positives}{correct\;positives+false\;positives}$$

(11) $$F1-Measure=\frac{2Precision\times Recall}{Precision+Recall}.$$

The total number of real positives is the number of all correct matches for the two keypoint sets. Correct positives, false positives and the total number of real positives are determined by using ground truth homography H, which can be obtained in two ways. For some datasets, the homography H between image pairs is given. The second method is that the homography H can be computed by manually selecting the corresponding points of the reference and the target images. For the set consisting of matching pairs obtained by the nearest neighbor distance ratio (NNDR) [3], we can calculate the projection error of every pair of points: if the error is less than some threshold, it is regarded as a correct positive. Similarly, the two point sets form a set consisting of all the possible matching pairs, the projection error of every pair of points in this set is computed, and if the error is less than the threshold, it will be regarded as a real positive. Thus, we can obtain the total number of real positives.

### 3.3. Parameter Settings

The local window size of the proposed descriptor was assigned to 80 × 80, the same as EOH, LGHD, and PCEHD. The local window sizes of other descriptors were set by default. We use $N_{s}=4$ and $N_{o}=6$ to express the number of convolution scales and orientations of the log-Gabor filter in the proposed descriptor. The projection error threshold was assigned to 5 for all three datasets. The threshold of matching method NNDR was varied from 0.8 to 1 in 10 equal steps.

### 3.4. Description of Compared Methods

In this subsection, we present a brief introduction of the methods compared in our experiments; they are summarized as follows:

(1) SIFT [3]: SIFT is the most widely used feature descriptor utilized in image registration and is based on the histogram of oriented gradients. The parameters in SIFT are set the same way as in [3].
(2) LGHD [31]: LGHD is a descriptor that directly utilizes the log-Gabor filter response as the constituent in the descriptor. In this study, a bank of 24 log-Gabor filters was utilized to represent filters of four scales and six orientations.
(3) EOH [16]: EOH is a descriptor that utilizes the results of the edge detector; this descriptor consists of the maximum response of the orientation filter on the results of the edge detector.
(4) PIIFD [13]: PIIFD is a partially intensity invariant feature descriptor that utilizes the image outlines within local windows to generate orientation histograms. We utilized the suggested parameters in this study.
(5) GDISIFT [14]: It constrains the range of the main orientation of each keypoint within [0, π] instead of [0, 2π] under the assumption that the non-linear intensity change causes the gradient orientation inverse in the opposite direction.
(6) HOG [9]: HOG is a feature descriptor used in computer vision; it counts occurrences of gradient orientation in localized portions of an image. HOG is computed on a dense grid of uniformly spaced cells and uses overlapping local contrast normalization for improved accuracy.
(7) SURF [6]: SURF is a fast and robust algorithm for tasks such as object recognition and image registration. Its descriptor is based on the sum of the Haar wavelet response around the keypoint.
(8) PCEHD [33]: PCEHD is a descriptor proposed for solving the correspondence problem between multimodal images. This descriptor is based on the combination of responses of log-Gabor filters and spatial information on the shape of the neighboring region around keypoints.

### 3.5. Performance Tests on Three Datasets and Analysis

In this subsection, we describe the testing of the proposed method on the three datasets and compare the results with those obtained using the other state-of-art descriptors mentioned above. We present the overall results of average precision, recall, and F-Measure on these datasets, and the Precision-Recall (PR) curves of some examples from each dataset are also illustrated.

Before we investigate the results in detail, we need to have an intuitive understanding of what a good PR curve should be like and how to obtain such a PR curve. A PR curve is obtained by varying the matching threshold. In our experiments, we utilized the NNDR [3] matching method to obtain the PR curve, in which the nearest and the second nearest distances between keypoints were calculated. Then, the ratio of the nearest and the second nearest distances was compared with a predefined threshold; this matching was rejected if the ratio was over the threshold. Therefore, by varying the threshold, we obtained different matching results for the same image pair. In our experiments, the threshold utilized in later experiments was varied from 0.8 to 1 in 10 equal steps. Therefore, for a pair of images for each method, there were 10 different precision values and recalls corresponding to these 10 thresholds. As the threshold increased, the ratio increased and more matching keypoint pairs were regarded as matches. Therefore, the recall should increase as the threshold becomes larger and the precision decreases. As a comprehensive result, the PR curve should have a decreasing trend for all the methods. Furthermore, a good method corresponds to a curve which is the most outside.

#### 3.5.1. Performance on Dataset 1

**Figure 3:**
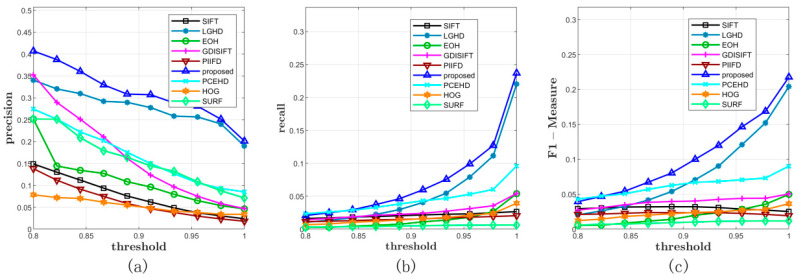
Comprehensive results on Dataset 1. (**a**) and (**b**) represent the average precisions and recalls of all the methods under different thresholds. (**c**) illustrates the F-Measure curves.

**Figure 4:**
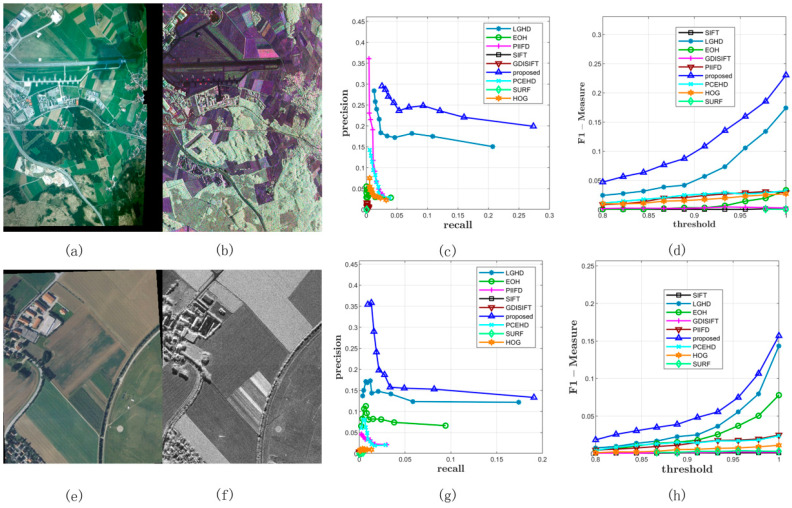
Two examples of results in Dataset 1. (**a**) and (**b**) represent the RGB image and the LiDAR intensity image, respectively. (**c**) represents the PR curves obtained from this image pair. (**d**) illustrates the F-Measure curves. (**e**–**h**) are similar with the first row.

We verified the performance of the proposed method on Diaz’s dataset first, which contained 15 pairs of images. By varying the NNDR matching threshold from 0.8 to 1 with a constant step width, we obtained 10 precisions and recalls on each pair of images. In the later experiments on Dataset 2 and Dataset 3, the configurations of the matching method and the matching threshold, i.e., NNDR and the domain of the threshold, were kept the same as the configurations in Dataset 1. For experiments on Dataset 1, the comprehensive results are shown in Figure 3 and Table 1. First of all, in terms of precision, the blue curve, which corresponds to the proposed method, was over all the other curves. The LGHD method and the PCEHD method ranked second and third, respectively. The performances of the SIFT, HOG, SURF, PIIFD, and EOH methods were similar, and their maximum average precision on the varying threshold was not over 0.3. In terms of recall, as is shown in Figure 3b, the blue curve was over all the other curves, which indicated that the proposed method outperformed the other methods in terms of recall. The LGHD method exhibited lower performance than the proposed method and ranked second. Another phase congruency-based method PCEHD exhibited similar performance to that of the proposed descriptor and LGHD when the threshold was low, but its recall increased slowly with an increase in the threshold. The curves of the remaining methods almost overlapped and were below the curves of the proposed method, LGHD and PCEHD, which indicated that the corresponding methods had similar performances in terms of recall and were not comparable to the proposed descriptor, LGHD and PCEHD. Considering the results on precision and recall together, which are shown in Figure 3c, we concluded that the proposed descriptor outperformed the other methods in terms of precision and recall; two examples from Dataset 1 are shown in Figure 4.

**Figure 5:**
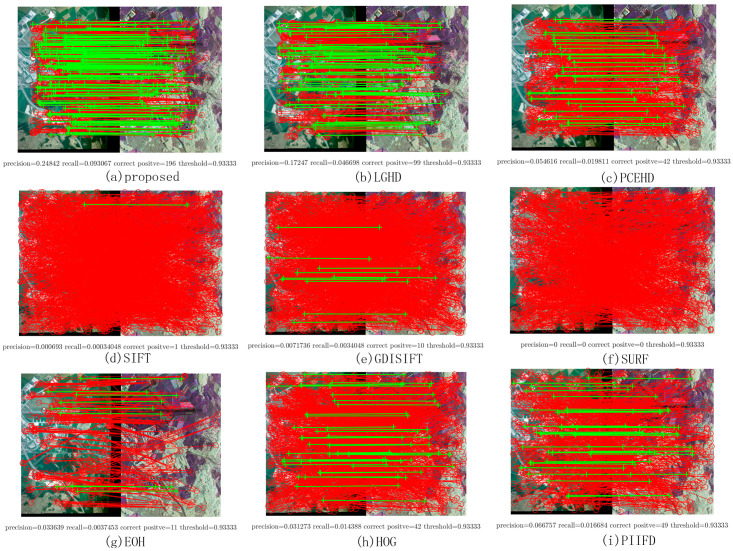
Matching results of one image pair from Dataset 1. The red solid line represents the matches obtained by using the nearest neighbor distance ratio (NNDR) method. Green lines are the correct positives.

Two image pairs from Dataset 1 were chosen to show the matching results of each method; they are shown in Figure 4. The left most image in each row represents the visible image, Figure 4b,f represents the LiDAR intensity images, and the figures on the right represent the PR curve and the F-Measure. As mentioned above, the PR curve that is the most outside indicates that this method has better performance. Thus, from Figure 4, it was obvious that the proposed method significantly outperformed the other methods, and the LGHD method yielded a relatively good result but was not comparable to the proposed method. All the remaining methods did not perform well, and the SIFT, GDISIFT, and SURF methods failed completely in both the cases. Specific matching results are shown in Figure 5; the correct matches are connected by green lines, and all the correspondences obtained are connected by red lines. The results in Figure 5 correspond to the seventh points in the PR curves in Figure 4c, in which the matching threshold is set to 0.933, as shown in Figure 5. It can be seen that the proposed method and the LGHD method achieved the two best results. Furthermore, the proposed method outperformed the LGHD in terms of precision, recall, and correct positives. The proposed method not only yielded the largest number of correct positives but also generated the highest precision and recall among all the considered methods. The number of correct positives (196 correct positives) was nearly twice that of the LGHD method (99 correct positives), as shown in Figure 5a,b.

#### 3.5.2. Performance on Dataset 2

**Figure 6:**
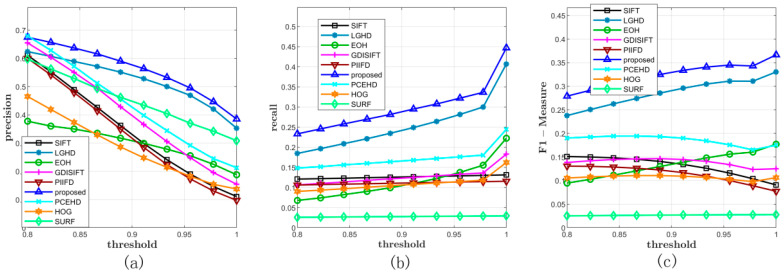
Comprehensive results on Dataset 2. (**a**,**b**) represent the average precisions and recalls of all the methods under different thresholds. (**c**) illustrates the F-Measure curves.

**Figure 7:**
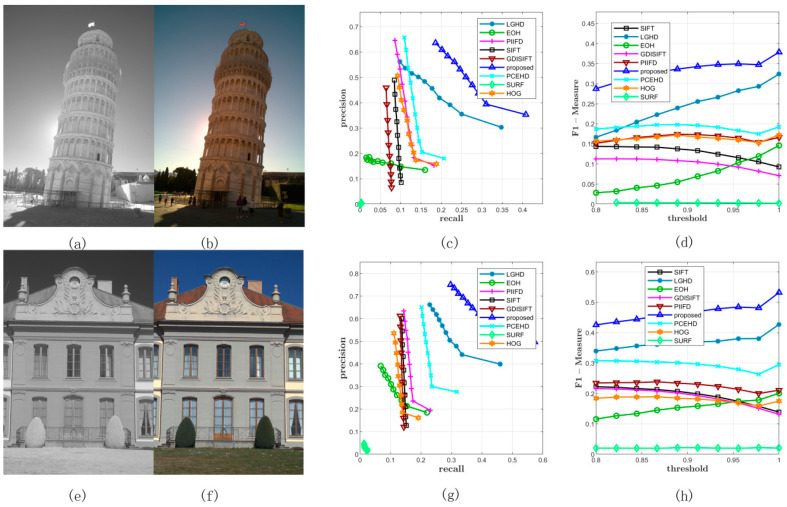
Two examples from Dataset 2. (**a**,**b**) represent the infrared and the RGB image respectively, which is a scene of a tower. (**c**) represents the PR curves obtained from this image pair. (**d**) illustrates the F-Measure curves. (**e**–**h**) are similar with the first row.

The comprehensive matching results of Dataset 2 are illustrated in Figure 6 and Table 2. All the methods were tested on 477 image pairs from 9 different categories of scenes. Figure 6 shows the average precision, recall, and F-Measure on varying NNDR thresholds. It was obvious that the proposed method had the best performance in terms of precision and recall; the LGHD method had the second best performance, in which the curves of LGHD in terms of recall and precision were completely below the curves of the proposed method. The PCEHD method ranked the third, and the remaining methods did not perform well with relatively low recall values for all the varying matching thresholds. In Figure 6a, all the methods seemed to have satisfactory precision results when the threshold was 0.8, and the average precisions of PCEHD and GDISIFT were comparable to the average precision of the proposed method at this time. Once the threshold was over 0.8, the curves of PCEHD, HOG, SURF, SIFT, EOH, PIIFD, and GDISIFT dropped quickly; however, the curves of the proposed method and LGHD dropped relatively slowly. Even though the precisions of SIFT, GDISIFT, PCEHD, SURF, and PIIFD at first were relatively high, the extremely low recall values of the methods made their relatively high precision values useless. Therefore, to draw a comprehensive conclusion, we checked the results of the F-Measure shown in Figure 6c; here, a higher value indicated better comprehensive performance. According to the results in Figure 6c, the proposed method outperformed the other methods in terms of precision and recall in the case of Dataset 2. Two examples are also illustrated in Figure 7.

**Figure 8:**
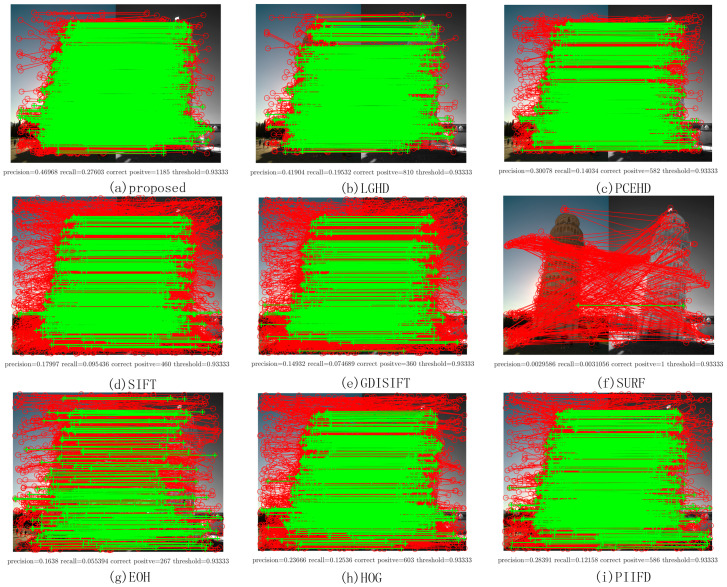
Matching results of one image pair from Dataset 2. The red solid line represents the matches obtained by using the NNDR method. Green lines are the correct positives.

Figure 7 shows two examples from Dataset 2; the left most image in the first column is captured by the near infrared band, the images in the second column are RGB images, and the figures in the third column and the fourth column represent the PR curves and the F-Measure curves of two image pairs, respectively. As shown in Figure 7c,d,g,h, the blue curve that stands for the proposed method was plotted the most outside, which meant that for both the image pairs, the proposed method outperformed the other methods in terms of precision and recall. For the SIFT, GDISIFT, HOG, and PIIFD methods, they have a common idea that the local descriptor is formed by the orientation histogram in local window; therefore, if the orientation is not defined or aligned delicately and correctly, the subsequent orientation histogram will not yield a satisfactory result. We can see that at the beginning of the PR curves, their precision was relatively very high, but their high precision was achieved at a very low recall, which implied that very few keypoints were identified as matches; therefore, a small number of correct matches could still yield a very high precision, which made no sense for practical matching. Moreover, because of their poor discriminative ability, their precision dropped very quickly, which was consistent with the results shown in Figure 6a. Figure 8 shows the specific matching results of the first image pair in Figure 7, which corresponded to the seventh points in the PR curves of all the methods shown in Figure 7c. It can be seen that the proposed method achieved the best performance in terms of precision, recall, and number of correct positives. The proposed method and the LGHD obtained more correct positives than the other methods, both of which were over 800: the proposed method obtained 1185 correct positives, and the LGHD obtained 810 correct positives.

#### 3.5.3. Performance on Dataset 3

**Figure 9:**
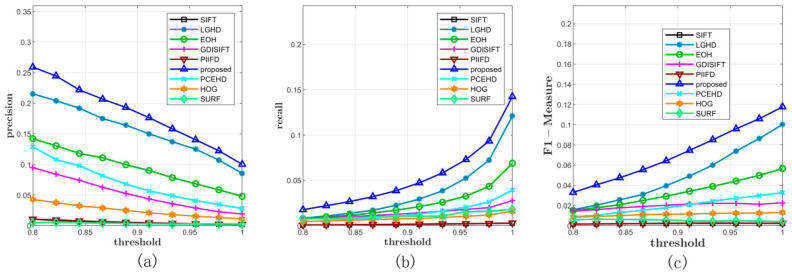
Comprehensive results on Dataset 3. (**a**,**b**) represent the average precisions and recalls of all the methods under different thresholds. (**c**) illustrates the F-Measure curves.

**Figure 10:**
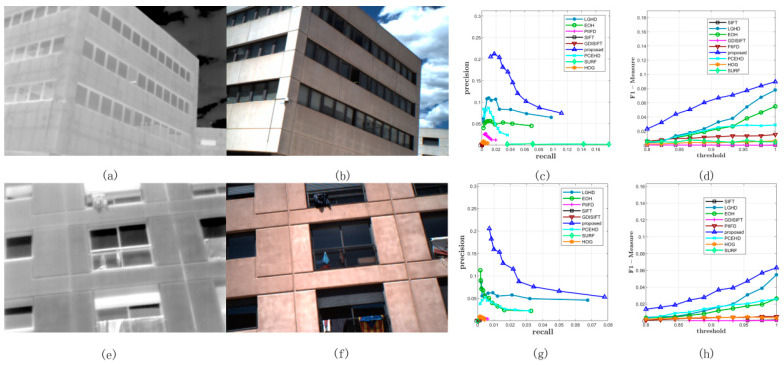
Two examples from Dataset 3. (**a**,**b**) represent the long wave infrared (LWIR) and RGB image respectively. (**c**) represents the PR curves obtained from this image pair. (**d**) illustrates the F-Measure curves. (**e**–**h**) are similar with the first row.

**Figure 11:**
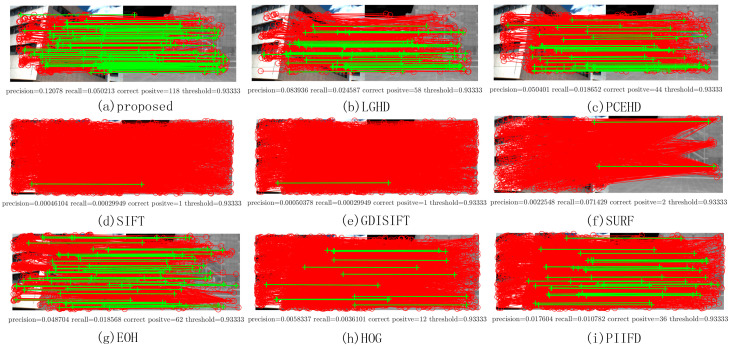
Matching results of one image pair from Dataset 3. The red solid line represents the matches obtained by using the NNDR method. Green lines are the correct positives.

All the methods were tested on Dataset 3 with a varying matching threshold. This dataset consisted of two parts: 44 RGB–LWIR image pairs with a resolution of 639 × 431 and 100 visible–LWIR image pairs with a resolution of 506 × 408. The configurations of the matching method and the threshold were the same as those of the former experiments. The average precision, recall, and F-Measure curves on this dataset are shown in Figure 9 and Table 3. In terms of precision, the proposed method outperformed the other eight methods; the LGHD method ranked the second, as shown in Figure 9a. The SIFT, SURF, and GDISIFT methods had similar performance, and all of them yielded unsatisfactory precision results. In terms of recall, the blue curve that corresponds to the proposed method was over the other eight curves. The recall value of the proposed method increased the fastest with an increase in the threshold. The LGHD and EOH methods had lower recall values and ranked second and third, respectively. The recall values of the SIFT, PIIFD, and SURF methods were considerably lower, and the recall curves of these methods were nearly horizontal. In summary, according to the results shown in Figure 9c, which represents the F-Measure curves indicating the comprehensive performance of all the methods, the proposed method outperformed the other methods in terms of the precision and recall for Dataset 3. Two specific matching examples are shown in Figure 10 and Figure 11.

Figure 10 shows two examples of matching results on Dataset 3: the images in the first column represent LWIR images, and the images in the second column are RGB images. Figure 10c,g illustrates the PR curves, and Figure 10d,h illustrates the F-Measure curves. Compared with the PR curves in the former datasets, the results of all the methods on Dataset 3 were not comparable to those of the former ones; the recall values of all the methods in both the examples could not reach 0.2 with a varying matching threshold, as shown in Figure 10c,g. This was mainly because the long wave infrared band is far away from the visible band. Even though it was not easy to match features in LWIR-RGB cases, we observed that the proposed method still yielded better results than the other methods and the F-Measure curves of the proposed method were over those of the other methods. Figure 11 shows the specific matching results of the first image pair in Figure 10, which corresponded to the seventh points in the PR curves shown in Figure 10c. It was obvious that the proposed method achieved the highest performance of all the methods in terms of precision, recall, and correct positives. The LGHD yielded the second best result, but the number of correct positives of the proposed method was nearly twice that of LGHD.

### 3.6. Computational Time Analysis

**Figure 12:**
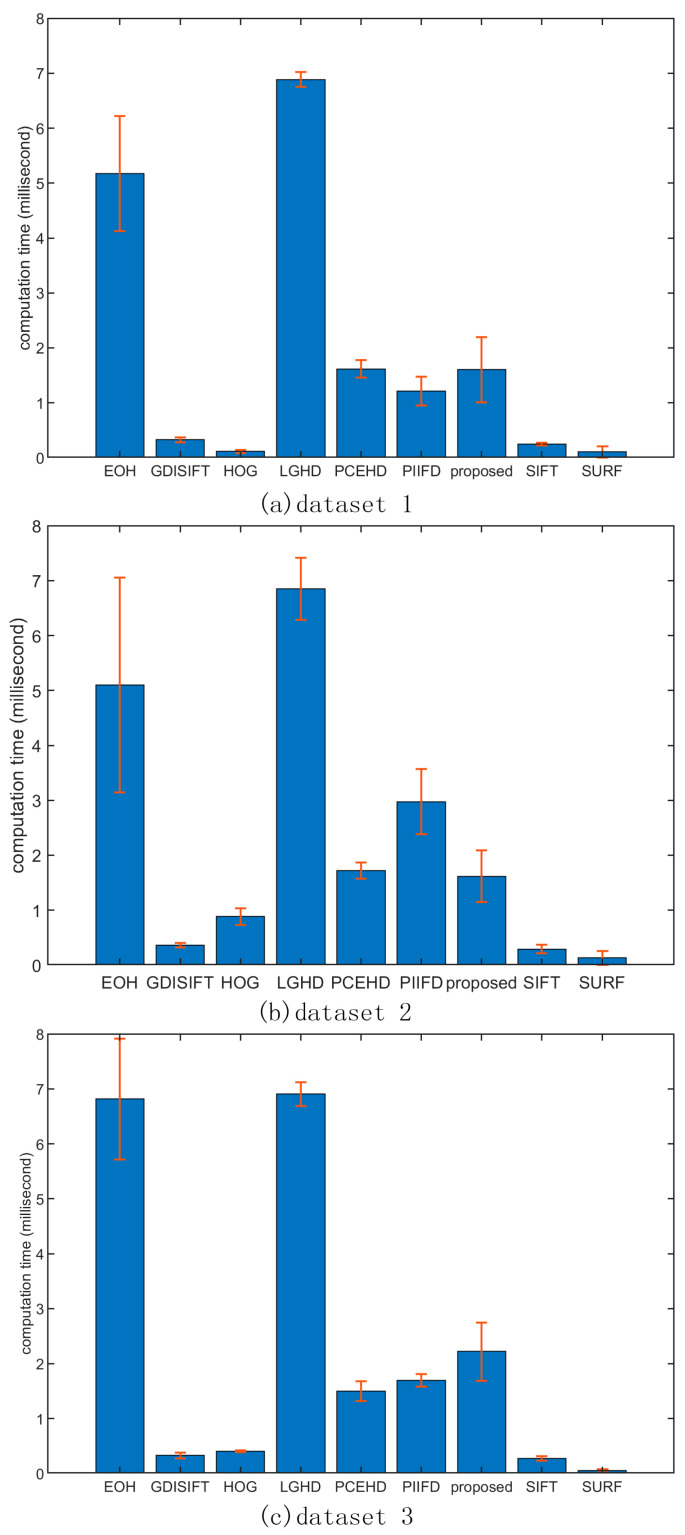
Error bar of each descriptor for each feature point on three datasets. The unit of y-axis is in milliseconds per descriptor.

All the experimental results were obtained using a PC with an Intel Core i7 CPU @ 2.8 GHz and 24 GB RAM. The average computational times of the descriptors for each feature point of each dataset are shown in Figure 12. It was obvious that on all three datasets, the LGHD and EOH methods were the two most time-consuming methods; the average computational times of the PCEHD, PIIFD, and proposed methods were similar for these three datasets and were considerably lower than those of LGHD and EOH. The computational times of HOG, SURF, GDISIFT, and SIFT were far lower than those of the methods mentioned above. As to the LGHD method, it divides the local window into 4 × 4 blocks, in which each block forms a 24-bin histogram. Therefore, the total size of the LGHD descriptor is 384. Thus, its average time of descriptor generation was the highest because of its highest descriptor dimension. The EOH method needs to extract the Canny edges for each local window before encoding its descriptor, which is time consuming even though its descriptor dimension is only 80. Therefore, it was the second most time-consuming method. The PCEHD and the proposed method are both phase congruency-based methods, and their descriptor generation needs the Fourier transform, inverse Fourier transform of an image, and convolution of an image with a 4 × 6 log-Gabor filter bank, which is time consuming. Compared with LGHD and EOH, the PCEHD and the proposed methods do not need to extract edges for every local window, and their descriptor dimensions are not high; therefore, both of them were faster than EOH and LGHD. HOG, SIFT, PIIFD, and GDISIFT are oriented gradient-based methods, which need to obtain the orientation of each keypoint. However, in the case of PIIFD, the descriptor generation involves large matrix operation, which will take considerably more time than GDISIFT, HOG, and SIFT. The SURF descriptor is obtained based on the sum of the Haar wavelet response around each keypoint, which is also fast. In summary, in terms of the average descriptor generation time of each method for the three datasets, the proposed method yielded superior results to those of EOH and LGHD; the average computational time of the proposed method was similar with that of PCEHD and PIIFD but was higher than that of SIFT, GDISIFT, HOG, and SURF, whose descriptor generation time was within 1 ms/descriptor.

## 4. Discussion

As discussed above, the experiments in our paper were carried out on three datasets, and the average performance evaluation results on each dataset and the specific matching results are both presented. The F-Measure curves of the proposed method were always over the curves of the other eight methods, which indicated that it had the best performance in terms of precision and recall. The proposed descriptor contains two parts. The first part is the oriented magnitude histogram, which can indicate the distribution of magnitude along different orientations. This part is similar to the generation in the LGHD method, but the LGHD descriptor has a considerably higher descriptor dimension (the descriptor size is 384). Therefore, the LGHD method exhibited the second best performance in most cases but with considerably more time. The second part of the descriptor is based on the principal axis orientation, in which the principal axis corresponds to the axis about which the moment of phase congruency is minimized. Furthermore, the minimized moment of phase congruency is the indication of feature points, which is assumed to be maintained in a multimodal image pair. Thus, the principal axis orientation-based histogram contributed to the superior performance as compared to the conventional oriented gradient-based descriptors, i.e., SIFT, HOG, and GDISIFT. The GDISIFT method modified the process of orientation assignment by directly inversing the orientation calculated in SIFT; thus, it had a similar performance to that of SIFT. The EOH method generates the descriptor under the assumption that the outline in multimodal images can be maintained after edge extraction, but its limited performance implied that EOH failed in most cases because of the poor results of the Canny algorithm. PCEHD is also a phase congruency-based descriptor, it combines the log-Gabor filter responses and spatial information on the shape of the local window; thus, it had better performance than the gradient-based methods. The SURF descriptor is generated based on the Haar wavelet responses on integral images. Those responses are vulnerable to intensity distortions; therefore, the SURF descriptor performed the worst in the cases of all the three datasets.

In terms of the average computational time of each method, SIFT, HOG, SURF, and GDISIFT significantly outperformed the other methods, and the computational times of the proposed descriptor, PCEHD, and PIIFD were higher than those of the gradient-based methods mentioned above. EOH and LGHD had a relatively high computational cost, and LGHD was the most time-consuming method among all the considered methods.

To draw a comprehensive conclusion, the proposed method outperformed the other methods, including the classic and the state-of-art feature descriptors, in terms of the recall and the precision within an acceptable computational time.

## 5. Conclusions

In this paper, a new descriptor based on the oriented magnitude histogram and the orientation of the minimum moment of phase congruency was proposed to tackle the problem of non-linear intensity changes in multimodal image registration. Experiments on three datasets were conducted, in which RGB–LiDAR, RGB–infrared, and RGB–LWIR image pairs were included. The experimental results proved that the proposed method outperformed most of the classic and the state-of-art descriptors in terms of the precision and the recall with an acceptable computational time cost. Considering the illumination and contrast invariant properties of phase congruency, some intermediate metrics obtained in this process can be further investigated to improve the performance of the proposed descriptor in terms of feature matching, which can be a future work.

## Figures and Tables

**Table 1:** Average precision, recall, and F-Measure of each method under different thresholds on Dataset 1.

		Threshold									
		0.8	0.822	0.844	0.867	0.889	0.911	0.933	0.956	0.978	1
**SIFT**	Precision	0.149	0.13	0.112	0.094	0.076	0.062	0.049	0.038	0.03	0.024
	Recall	0.016	0.017	0.018	0.019	0.02	0.022	0.023	0.023	0.025	0.027
	F-Measure	0.029	0.03	0.032	0.032	0.032	0.032	0.031	0.029	0.027	0.025
**LGHD**	Precision	0.341	0.321	0.31	0.292	0.29	0.278	0.258	0.257	0.24	0.19
	Recall	0.011	0.014	0.017	0.022	0.03	0.041	0.055	0.079	0.111	0.22
	F-Measure	0.02	0.026	0.033	0.042	0.054	0.071	0.09	0.121	0.152	0.204
**EOH**	Precision	0.252	0.145	0.134	0.127	0.109	0.096	0.08	0.065	0.054	0.046
	Recall	0.003	0.003	0.004	0.006	0.008	0.011	0.014	0.018	0.027	0.054
	F-Measure	0.006	0.006	0.009	0.012	0.014	0.02	0.024	0.029	0.036	0.05
**GDISIFT**	Precision	0.352	0.29	0.251	0.211	0.162	0.124	0.097	0.075	0.059	0.047
	Recall	0.014	0.016	0.019	0.021	0.023	0.024	0.027	0.031	0.036	0.054
	F-Measure	0.027	0.031	0.035	0.038	0.04	0.04	0.043	0.044	0.044	0.05
**PIIFD**	Precision	0.138	0.112	0.091	0.075	0.059	0.047	0.038	0.03	0.024	0.018
	Recall	0.011	0.012	0.013	0.014	0.015	0.016	0.017	0.018	0.019	0.02
	F-Measure	0.021	0.022	0.023	0.024	0.024	0.024	0.024	0.023	0.021	0.019
**Proposed**	Precision	0.407	0.387	0.36	0.33	0.309	0.307	0.289	0.281	0.251	0.201
	Recall	0.021	0.025	0.029	0.037	0.046	0.06	0.076	0.099	0.127	0.237
	F-Measure	0.039	0.047	0.054	0.067	0.08	0.1	0.12	0.147	0.169	0.218
**PCEHD**	Precision	0.274	0.251	0.222	0.202	0.176	0.15	0.127	0.106	0.093	0.085
	Recall	0.023	0.026	0.029	0.033	0.038	0.043	0.047	0.053	0.06	0.096
	F-Measure	0.043	0.047	0.051	0.057	0.063	0.067	0.068	0.071	0.073	0.09
**HOG**	Precision	0.079	0.072	0.07	0.062	0.055	0.048	0.041	0.038	0.034	0.034
	Recall	0.007	0.008	0.011	0.012	0.014	0.016	0.018	0.021	0.024	0.039
	F-Measure	0.012	0.014	0.018	0.02	0.022	0.024	0.025	0.027	0.028	0.037
**SURF**	Precision	0.252	0.251	0.209	0.179	0.164	0.145	0.132	0.108	0.088	0.071
	Recall	0.003	0.004	0.004	0.004	0.005	0.005	0.006	0.006	0.006	0.006
	F-Measure	0.006	0.007	0.008	0.008	0.009	0.01	0.011	0.012	0.012	0.012

**Table 2:** Average precision, recall, and F-Measure of each method under different thresholds on Dataset 2.

		Threshold									
		0.8	0.822	0.844	0.867	0.889	0.911	0.933	0.956	0.978	1
**SIFT**	Precision	0.508	0.454	0.397	0.341	0.285	0.232	0.185	0.145	0.111	0.084
	Recall	0.089	0.09	0.091	0.092	0.093	0.094	0.096	0.097	0.098	0.099
	F-Measure	0.151	0.15	0.148	0.145	0.141	0.134	0.126	0.116	0.104	0.091
**LGHD**	Precision	0.546	0.533	0.517	0.501	0.483	0.462	0.439	0.409	0.367	0.307
	Recall	0.152	0.164	0.176	0.189	0.203	0.217	0.233	0.251	0.269	0.357
	F-Measure	0.238	0.251	0.263	0.274	0.286	0.296	0.305	0.311	0.311	0.33
**EOH**	Precision	0.33	0.316	0.305	0.292	0.277	0.261	0.242	0.22	0.193	0.164
	Recall	0.055	0.061	0.068	0.076	0.085	0.095	0.107	0.121	0.137	0.193
	F-Measure	0.095	0.103	0.112	0.121	0.13	0.139	0.148	0.156	0.16	0.177
**GDISIFT**	Precision	0.561	0.517	0.468	0.414	0.358	0.301	0.246	0.195	0.15	0.116
	Recall	0.079	0.082	0.086	0.089	0.092	0.095	0.099	0.102	0.105	0.136
	F-Measure	0.139	0.142	0.145	0.146	0.147	0.145	0.141	0.134	0.124	0.125
**PIIFD**	Precision	0.494	0.438	0.38	0.321	0.266	0.213	0.166	0.128	0.096	0.072
	Recall	0.075	0.076	0.078	0.079	0.08	0.081	0.081	0.082	0.083	0.084
	F-Measure	0.131	0.13	0.129	0.126	0.123	0.117	0.109	0.1	0.089	0.077
**Proposed**	Precision	0.584	0.569	0.554	0.536	0.517	0.495	0.47	0.439	0.396	0.341
	Recall	0.183	0.196	0.209	0.223	0.237	0.252	0.267	0.284	0.302	0.397
	F-Measure	0.279	0.292	0.304	0.315	0.325	0.334	0.341	0.345	0.343	0.367
**PCEHD**	Precision	0.602	0.553	0.503	0.448	0.393	0.338	0.286	0.237	0.194	0.163
	Recall	0.113	0.117	0.121	0.124	0.128	0.132	0.136	0.14	0.144	0.189
	F-Measure	0.19	0.193	0.194	0.195	0.193	0.19	0.184	0.176	0.165	0.175
**HOG**	Precision	0.368	0.329	0.289	0.253	0.218	0.187	0.159	0.134	0.112	0.099
	Recall	0.062	0.065	0.068	0.071	0.074	0.077	0.081	0.084	0.087	0.116
	F-Measure	0.106	0.108	0.11	0.111	0.111	0.109	0.107	0.103	0.098	0.106
**SURF**	Precision	0.514	0.474	0.435	0.398	0.361	0.325	0.292	0.261	0.234	0.207
	Recall	0.013	0.013	0.014	0.014	0.014	0.014	0.014	0.015	0.015	0.015
	F-Measure	0.025	0.026	0.026	0.027	0.027	0.027	0.027	0.028	0.028	0.028

**Table 3:** Average precision, recall, and F-Measure of each method under different thresholds on Dataset 3.

		Threshold									
		0.8	0.822	0.844	0.867	0.889	0.911	0.933	0.956	0.978	1
**SIFT**	Precision	0.011	0.009	0.007	0.006	0.005	0.004	0.003	0.003	0.002	0.002
	Recall	0.001	0.001	0.001	0.001	0.001	0.002	0.002	0.002	0.002	0.003
	F-Measure	0.002	0.002	0.002	0.002	0.002	0.002	0.002	0.002	0.002	0.002
**LGHD**	Precision	0.215	0.204	0.192	0.175	0.164	0.15	0.137	0.125	0.107	0.086
	Recall	0.008	0.011	0.014	0.017	0.023	0.029	0.038	0.052	0.072	0.121
	F-Measure	0.016	0.02	0.026	0.031	0.04	0.049	0.06	0.074	0.086	0.1
**EOH**	Precision	0.142	0.131	0.118	0.111	0.1	0.09	0.078	0.068	0.059	0.048
	Recall	0.008	0.01	0.011	0.014	0.017	0.021	0.026	0.033	0.043	0.069
	F-Measure	0.015	0.018	0.021	0.025	0.029	0.034	0.039	0.044	0.05	0.057
**GDISIFT**	Precision	0.095	0.084	0.074	0.063	0.053	0.044	0.036	0.029	0.023	0.019
	Recall	0.008	0.009	0.01	0.011	0.013	0.014	0.016	0.018	0.02	0.028
	F-Measure	0.014	0.016	0.018	0.019	0.02	0.021	0.022	0.022	0.021	0.023
**PIIFD**	Precision	0.011	0.01	0.008	0.007	0.006	0.005	0.004	0.003	0.002	0.002
	Recall	0.001	0.001	0.001	0.001	0.002	0.002	0.002	0.002	0.003	0.003
	F-Measure	0.001	0.002	0.002	0.002	0.002	0.003	0.003	0.003	0.003	0.002
**Proposed**	Precision	0.259	0.244	0.222	0.207	0.193	0.176	0.158	0.14	0.122	0.1
	Recall	0.018	0.022	0.027	0.032	0.039	0.047	0.058	0.073	0.093	0.142
	F-Measure	0.033	0.04	0.047	0.055	0.064	0.075	0.085	0.096	0.106	0.118
**PCEHD**	Precision	0.129	0.108	0.098	0.082	0.068	0.057	0.049	0.041	0.035	0.028
	Recall	0.005	0.005	0.007	0.008	0.01	0.013	0.016	0.02	0.026	0.039
	F-Measure	0.009	0.01	0.013	0.015	0.018	0.021	0.024	0.027	0.03	0.033
**HOG**	Precision	0.043	0.038	0.032	0.029	0.025	0.021	0.018	0.016	0.013	0.011
	Recall	0.005	0.005	0.006	0.007	0.007	0.008	0.009	0.01	0.012	0.016
	F-Measure	0.008	0.009	0.01	0.011	0.011	0.012	0.012	0.012	0.012	0.013
**SURF**	Precision	0.005	0.006	0.005	0.004	0.004	0.003	0.003	0.003	0.003	0.002
	Recall	0.008	0.008	0.008	0.01	0.01	0.011	0.011	0.015	0.016	0.018
	F-Measure	0.006	0.006	0.006	0.006	0.006	0.005	0.004	0.005	0.005	0.004
